# 2-(Adamantan-1-yl)-*N*-(6-meth­oxy-1,3-benzo­thia­zol-2-yl)acetamide

**DOI:** 10.1107/S1600536813023313

**Published:** 2013-08-23

**Authors:** Alexander S. Bunev, Prokofij V. Sklyuev, Gennady I. Ostapenko, Petr P. Purygin, Victor N. Khrustalev

**Affiliations:** a Department of Chemistry and Chemical Technology, Togliatti State University, 14 Belorusskaya St, Togliatti 445667, Russian Federation; b Department of Organic, Bioorganic and Medicinal Chemistry, Samara State University, 1 Academician Pavlov St, Samara 443011, Russian Federation; c X-Ray Structural Centre, A.N. Nesmeyanov Institute of Organoelement Compounds, Russian Academy of Sciences, 28 Vavilov Street, B-334, Moscow 119991, Russian Federation

## Abstract

The asymmetric unit of the title compound, C_20_H_24_N_2_O_2_S, contains two independent mol­ecules having very similar geometries. The main *N*-(6-meth­oxy-1,3-benzo­thia­zol-2-yl)acetamide moiety adopts an almost planar structure (r.m.s. deviations of 0.091 and 0.051 Å for the two independent molecules). The adamantyl substituent occupies the *gauche* position relative to the C—N bond of the acetamide moiety [the corresponding N–C–C–C dihedral angles are −100.3 (3) and −96.5 (3)° for the two independent mol­ecules]. In the crystal, the two independent mol­ecules form a dimer *via* a pair of N—H⋯N hydrogen bonds. The dimers are further linked by C—H⋯O hydrogen bonds and attractive S⋯S [3.622 (2) Å] inter­actions into ribbons along [100].

## Related literature

For properties of benzo­thia­zoles as building blocks in organic synthesis, see: Gupta & Rawat (2010 ▶); Facchinetti *et al.* (2012 ▶); Sareen *et al.* (2012 ▶); Radatz *et al.* (2013 ▶). For syntheses and properties of 2-substituted benzo­thia­zoles, see: Hussein *et al.* (2012 ▶); Ugale *et al.* (2012 ▶); Yoo *et al.* (2012 ▶); Zhu *et al.* (2012 ▶); Bhardwaj *et al.* (2013 ▶); Patel *et al.* (2013 ▶).
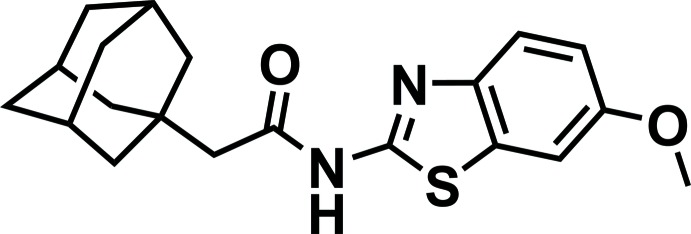


## Experimental

### 

#### Crystal data

C_20_H_24_N_2_O_2_S
*M* *_r_* = 356.48
Triclinic, 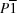

*a* = 11.0114 (13) Å
*b* = 13.6647 (18) Å
*c* = 13.9230 (18) Å
α = 61.554 (3)°
β = 80.252 (3)°
γ = 89.782 (4)°
*V* = 1808.3 (4) Å^3^
*Z* = 4
Mo *K*α radiation
μ = 0.20 mm^−1^
*T* = 100 K
0.20 × 0.15 × 0.10 mm

#### Data collection

Bruker APEXII CCD diffractometer
Absorption correction: multi-scan (*SADABS*; Bruker, 2003 ▶) *T*_min_ = 0.962, *T*_max_ = 0.981
17897 measured reflections
7124 independent reflections
4424 reflections with *I* > 2σ(*I*)
*R*_int_ = 0.071

#### Refinement

*R*[*F* ^2^ > 2σ(*F* ^2^)] = 0.049
*wR*(*F* ^2^) = 0.115
*S* = 0.91
7124 reflections
459 parameters
H atoms treated by a mixture of independent and constrained refinement
Δρ_max_ = 0.40 e Å^−3^
Δρ_min_ = −0.37 e Å^−3^

### 

Data collection: *APEX2* (Bruker, 2005 ▶); cell refinement: *SAINT* (Bruker, 2001 ▶); data reduction: *SAINT*; program(s) used to solve structure: *SHELXTL* (Sheldrick, 2008 ▶); program(s) used to refine structure: *SHELXTL*; molecular graphics: *SHELXTL*; software used to prepare material for publication: *SHELXTL*.

## Supplementary Material

Crystal structure: contains datablock(s) global, I. DOI: 10.1107/S1600536813023313/rk2412sup1.cif

Structure factors: contains datablock(s) I. DOI: 10.1107/S1600536813023313/rk2412Isup2.hkl

Supplementary material file. DOI: 10.1107/S1600536813023313/rk2412Isup3.cml

Additional supplementary materials:  crystallographic information; 3D view; checkCIF report

## Figures and Tables

**Table 1:** Hydrogen-bond geometry (Å, °)

*D*—H⋯*A*	*D*—H	H⋯*A*	*D*⋯*A*	*D*—H⋯*A*
N2—H2*N*⋯N3	0.87 (3)	2.13 (3)	2.995 (3)	169 (2)
N4—H4*N*⋯N1	0.78 (3)	2.30 (3)	3.077 (3)	174 (2)
C6—H6⋯O3^i^	0.95	2.58	3.452 (3)	153
C26—H26⋯O1^ii^	0.95	2.45	3.392 (3)	174

Symmetry codes: (i) 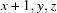; (ii) 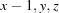.

**Fig. 1.**
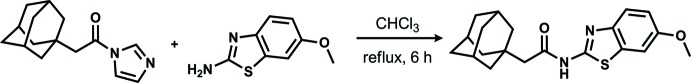
The reaction of 1-(1-adamantylacetyl)-1H-imidazole with 6-methoxy-1,3-benzothiazol-2-amine.

**Fig. 2.**
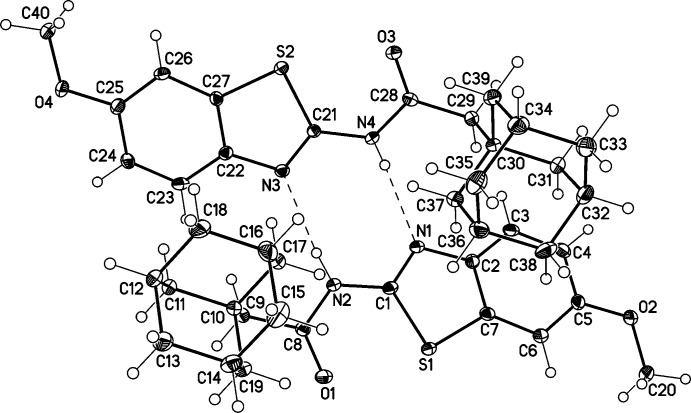
Molecular structure of I. The two crystallographically independent molecules forming the H-bonded dimer are shown. Displacement ellipsoids are presented at the 40% probability level. H atoms are depicted as small spheres of arbitrary radius. The dashed lines indicate the intermolecular N–H···N hydrogen bonds.

**Fig. 3.**
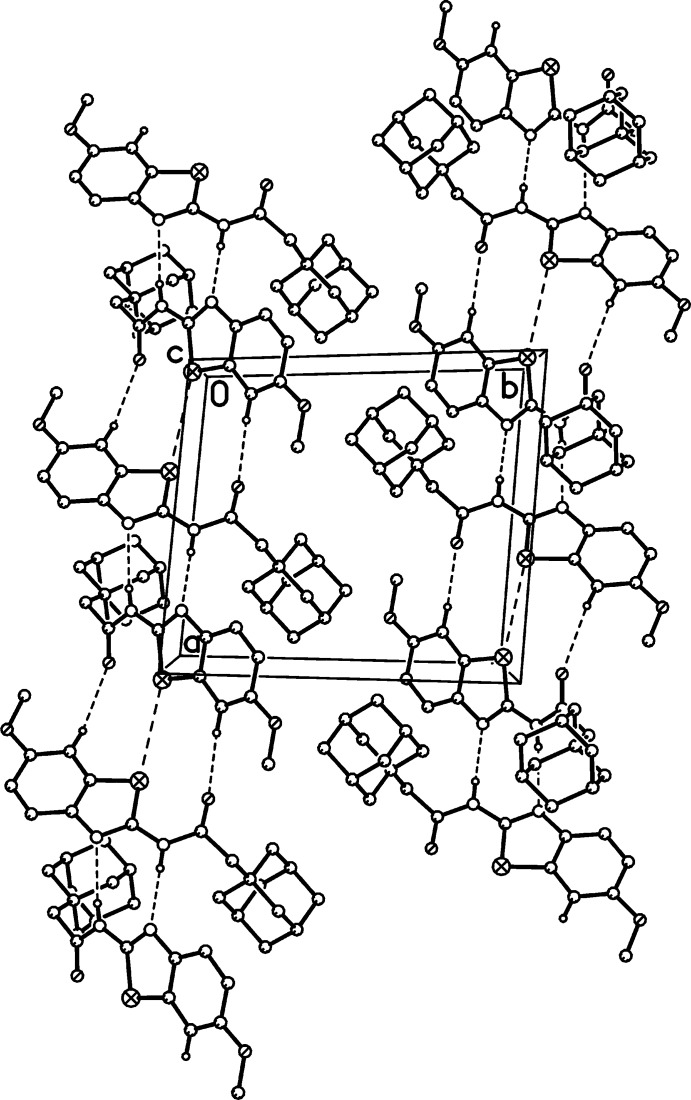
A portion of the crystal structure of I demonstrating the H-bonded ribbons toward [100]. The hydrogen atoms participating in the formation of hydrogen bonds are shown only. The intermolecular N–H···N and C–H···O hydrogen bonds as well as attractive S···S interactions are depicted by dashed lines.

## supplementary crystallographic information

### 1. Comment

Benzothiazoles are important and versatile building blocks in organic synthesis, in particular, for production of various biologically active compounds in medicinal and industrial fields (Gupta & Rawat, 2010; Facchinetti *et al.*, 2012; Sareen *et al.*, 2012; Radatz *et al.*, 2013). Notably, among all benzothiazole derivatives, 2-substituted benzothiazoles are of special interest due to their multiple applications as medicinal agents, agrochemicals, materials for chemical sensors *etc.* (Hussein *et al.*, 2012; Ugale *et al.*, 2012; Yoo *et al.*, 2012; Zhu *et al.*, 2012; Bhardwaj *et al.*, 2013; Patel *et al.*, 2013).

In this work, a 2-(1-adamantyl)-*N*-(6-methoxy-1,3-benzothiazol-2-yl)acetamide, C_20_H_24_N_2_O_2_S, (I) was prepared by the reaction of 1-(1-adamantylacetyl)-1*H*-imidazole with 6-methoxy-1,3-benzothiazol-2-amine (Fig. 1) and its structure was unambiguously established by the X-ray diffraction study.

Compound I crystallizes in the triclinic *P*1 space group with two crystallographically independent molecules forming an H-bonded dimer by the two classical intermolecular N–H···N hydrogen bonds (Table 1, Fig. 2). The geometries of these two independent molecules are very similar. The main *N*-(6-oxy-1,3-benzothiazol-2-yl)acetamide fragment adopts almost planar structure determined by the long chain of conjugated bonds. The adamantyl substituent occupies the *gauche* position in relative to the C–N bond of the acetamide moiety (the corresponding N–C–C–C dihedral angles are -100.3 (3)° and -96.5 (3)° for the two independent molecules, respectively).

In the crystal, the H-bonded dimers of I are linked by the intermolecular C6–H6···O3^i^ and C26–H26···O1^ii^ non-classical hydrogen bonds (Table 1) as well as attractive S1···S2^i^ (3.622 (2)Å) interactions into ribbons toward [100] (Fig. 3). Symmetry codes: (i) *x*+1, *y*, *z*; (ii) *x*-1, *y*, *z*.

### 2. Experimental

A mixture 1-(1-adamantylacetyl)-1*H*-imidazole (1.06 g, 4.3 mmol) and 6-methoxy-1,3-benzothiazol-2-amine (0.9 g, 4.9 mmol) in CHCl_3_ (50 ml) were refluxed for 6 h. The precipitate was filtered, and then reaction mixture was concentrated in *vacuo*. The residue crystallized from 80% *Et*OH. Yield is 22%. The single crystals of the product I was obtained by slow crystallization from *Et*OH. M.p. = 485-486 K. IR (KBr), ν/cm^-1^: 3178, 2903, 2848, 1668, 1604, 1472, 1267, 1062, 827. ^1^H NMR (500 MHz, DMSO-*d_6_*, 304 K): δ = 1.52-1.49 (m, 6H), 1.66-1.63 (m, 6H), 1.96-1.93 (m, 3H), 2.63-2.61(m, 2H), 3.76 (s, 3H), 7.03-7.01 (m, 1H), 7.35-7.34 (m, 1H), 7.73 (dd, 1H, *J* = 8.87). Anal. Calcd for C_20_H_24_N_2_O_2_S: C, 67.38; H, 6.79. Found: C,67.32; H, 6.82.

### 3. Refinement

The hydrogen atoms of the amino groups were localized in the difference Fourier map and included in the refinement with fixed positional and isotropic displacement parameters - *U*_iso_(H) = 1.2*U*_eq_(N). The other hydrogen atoms were placed in the calculated positions with C–H = 0.95Å (for aryl H), 0.98Å (for methyl H), 0.99Å (for methylene H), 1.00Å (for methine H) and refined in the riding model with fixed isotropic displacement parameters: *U*_iso_(H) = 1.5*U*_eq_(C) for the CH_3_ groups and 1.2*U*_eq_(C) for the other CH groups.

### Crystal data

**Table:**

C_20_H_24_N_2_O_2_S	*Z* = 4
*M**_r_* = 356.48	*F*(000) = 760
Triclinic, *P*1	*D*_x_ = 1.309 Mg m^−^^3^
Hall symbol: -P 1	Melting point = 485–486 K
*a* = 11.0114 (13) Å	Mo *K*α radiation, λ = 0.71073 Å
*b* = 13.6647 (18) Å	Cell parameters from 1305 reflections
*c* = 13.9230 (18) Å	θ = 2.8–24.8°
α = 61.554 (3)°	µ = 0.20 mm^−^^1^
β = 80.252 (3)°	*T* = 100 K
γ = 89.782 (4)°	Prism, colourless
*V* = 1808.3 (4) Å^3^	0.20 × 0.15 × 0.10 mm

### Data collection

**Table:**

Bruker APEXII CCD diffractometer	7124 independent reflections
Radiation source: fine-focus sealed tube	4424 reflections with *I* > 2σ(*I*)
Graphite monochromator	*R*_int_ = 0.071
φ and ω scans	θ_max_ = 26.0°, θ_min_ = 1.7°
Absorption correction: multi-scan (*SADABS*; Bruker, 2003)	*h* = −13→13
*T*_min_ = 0.962, *T*_max_ = 0.981	*k* = −16→16
17897 measured reflections	*l* = −17→17

### Refinement

**Table:**

Refinement on *F*^2^	Primary atom site location: structure-invariant direct methods
Least-squares matrix: full	Secondary atom site location: difference Fourier map
*R*[*F*^2^ > 2σ(*F*^2^)] = 0.049	Hydrogen site location: inferred from neighbouring sites
*wR*(*F*^2^) = 0.115	H atoms treated by a mixture of independent and constrained refinement
*S* = 0.91	*w* = 1/[σ^2^(*F*_o_^2^) + (0.0515*P*)^2^] where *P* = (*F*_o_^2^ + 2*F*_c_^2^)/3
7124 reflections	(Δ/σ)_max_ < 0.001
459 parameters	Δρ_max_ = 0.40 e Å^−^^3^
0 restraints	Δρ_min_ = −0.37 e Å^−^^3^

### Special details

**Table:**

Geometry. All s.u.'s (except the s.u. in the dihedral angle between two l.s. planes) are estimated using the full covariance matrix. The cell s.u.'s are taken into account individually in the estimation of s.u.'s in distances, angles and torsion angles; correlations between s.u.'s in cell parameters are only used when they are defined by crystal symmetry. An approximate (isotropic) treatment of cell s.u.'s is used for estimating s.u.'s involving l.s. planes.
Refinement. Refinement of *F*^2^ against ALL reflections. The weighted *R*-factor *wR* and goodness of fit *S* are based on *F*^2^, conventional *R*-factors *R* are based on *F*, with *F* set to zero for negative *F*^2^. The threshold expression of *F*^2^ > σ(*F*^2^) is used only for calculating *R*-factors(gt) *etc*. and is not relevant to the choice of reflections for refinement. *R*-factors based on *F*^2^ are statistically about twice as large as those based on *F*, and *R*-factors based on ALL data will be even larger.

### Fractional atomic coordinates and isotropic or equivalent isotropic displacement parameters (Å^2^)

**Table:**

	*x*	*y*	*z*	*U*_iso_*/*U*_eq_	
S1	0.64296 (6)	0.03880 (6)	0.19027 (6)	0.02258 (17)	
O1	0.59221 (16)	−0.16836 (15)	0.23783 (15)	0.0273 (4)	
O2	0.80076 (16)	0.44718 (15)	0.07773 (17)	0.0336 (5)	
N1	0.47389 (18)	0.14101 (17)	0.07440 (17)	0.0209 (5)	
N2	0.4527 (2)	−0.05386 (18)	0.14838 (18)	0.0211 (5)	
H2N	0.382 (2)	−0.052 (2)	0.128 (2)	0.025*	
C1	0.5129 (2)	0.0436 (2)	0.1310 (2)	0.0201 (6)	
C2	0.5505 (2)	0.2228 (2)	0.0741 (2)	0.0211 (6)	
C3	0.5429 (2)	0.3383 (2)	0.0151 (2)	0.0254 (6)	
H3	0.4801	0.3674	−0.0276	0.030*	
C4	0.6279 (2)	0.4093 (2)	0.0197 (2)	0.0291 (7)	
H4	0.6226	0.4877	−0.0197	0.035*	
C5	0.7219 (2)	0.3676 (2)	0.0817 (2)	0.0254 (6)	
C6	0.7324 (2)	0.2535 (2)	0.1401 (2)	0.0234 (6)	
H6	0.7962	0.2246	0.1817	0.028*	
C7	0.6449 (2)	0.1830 (2)	0.1350 (2)	0.0213 (6)	
C8	0.4930 (2)	−0.1576 (2)	0.2068 (2)	0.0205 (6)	
C9	0.4037 (2)	−0.2551 (2)	0.2358 (2)	0.0235 (6)	
H9A	0.4499	−0.3122	0.2243	0.028*	
H9B	0.3437	−0.2295	0.1851	0.028*	
C10	0.3327 (2)	−0.3087 (2)	0.3571 (2)	0.0219 (6)	
C11	0.2361 (2)	−0.3993 (2)	0.3743 (2)	0.0257 (6)	
H11A	0.1786	−0.3650	0.3219	0.031*	
H11B	0.2781	−0.4555	0.3583	0.031*	
C12	0.1631 (2)	−0.4565 (2)	0.4939 (2)	0.0303 (7)	
H12	0.1003	−0.5148	0.5035	0.036*	
C13	0.2510 (3)	−0.5111 (2)	0.5755 (2)	0.0320 (7)	
H13A	0.2927	−0.5690	0.5622	0.038*	
H13B	0.2036	−0.5475	0.6529	0.038*	
C14	0.3477 (2)	−0.4215 (2)	0.5595 (2)	0.0295 (7)	
H14	0.4058	−0.4571	0.6122	0.035*	
C15	0.2822 (3)	−0.3343 (3)	0.5832 (3)	0.0392 (8)	
H15A	0.3441	−0.2768	0.5741	0.047*	
H15B	0.2358	−0.3705	0.6608	0.047*	
C16	0.1931 (3)	−0.2794 (2)	0.5030 (2)	0.0347 (7)	
H16	0.1501	−0.2226	0.5190	0.042*	
C17	0.2657 (2)	−0.2223 (2)	0.3826 (2)	0.0278 (6)	
H17A	0.3270	−0.1635	0.3719	0.033*	
H17B	0.2082	−0.1866	0.3306	0.033*	
C18	0.0973 (2)	−0.3689 (2)	0.5174 (3)	0.0347 (7)	
H18A	0.0396	−0.3336	0.4655	0.042*	
H18B	0.0487	−0.4053	0.5943	0.042*	
C19	0.4204 (2)	−0.3648 (2)	0.4400 (2)	0.0246 (6)	
H19A	0.4636	−0.4210	0.4247	0.029*	
H19B	0.4836	−0.3079	0.4303	0.029*	
C20	0.9080 (2)	0.4100 (2)	0.1270 (2)	0.0304 (7)	
H20A	0.9542	0.4734	0.1230	0.046*	
H20B	0.9610	0.3784	0.0866	0.046*	
H20C	0.8822	0.3527	0.2050	0.046*	
S2	−0.03030 (6)	−0.00170 (5)	0.16571 (5)	0.02023 (16)	
O3	0.01738 (16)	0.16742 (15)	0.20276 (15)	0.0245 (4)	
O4	−0.15109 (16)	−0.34505 (15)	0.11643 (15)	0.0272 (4)	
N3	0.19426 (18)	−0.04250 (17)	0.10519 (16)	0.0190 (5)	
N4	0.18972 (19)	0.11000 (18)	0.13802 (18)	0.0205 (5)	
H4N	0.262 (2)	0.122 (2)	0.118 (2)	0.025*	
C21	0.1303 (2)	0.0230 (2)	0.1335 (2)	0.0173 (5)	
C22	0.1119 (2)	−0.1226 (2)	0.1075 (2)	0.0191 (6)	
C23	0.1451 (2)	−0.2080 (2)	0.0829 (2)	0.0230 (6)	
H23	0.2297	−0.2164	0.0625	0.028*	
C24	0.0539 (2)	−0.2798 (2)	0.0887 (2)	0.0225 (6)	
H24	0.0763	−0.3388	0.0733	0.027*	
C25	−0.0717 (2)	−0.2682 (2)	0.1169 (2)	0.0234 (6)	
C26	−0.1078 (2)	−0.1851 (2)	0.1433 (2)	0.0210 (6)	
H26	−0.1926	−0.1775	0.1641	0.025*	
C27	−0.0139 (2)	−0.1133 (2)	0.1380 (2)	0.0187 (5)	
C28	0.1302 (2)	0.1771 (2)	0.1762 (2)	0.0203 (6)	
C29	0.2130 (2)	0.2567 (2)	0.1874 (2)	0.0225 (6)	
H29A	0.1748	0.3282	0.1650	0.027*	
H29B	0.2932	0.2721	0.1360	0.027*	
C30	0.2369 (2)	0.2112 (2)	0.3078 (2)	0.0214 (6)	
C31	0.3265 (2)	0.2979 (2)	0.3073 (2)	0.0269 (6)	
H31A	0.4045	0.3099	0.2544	0.032*	
H31B	0.2892	0.3701	0.2825	0.032*	
C32	0.3540 (3)	0.2569 (3)	0.4246 (2)	0.0321 (7)	
H32	0.4122	0.3137	0.4233	0.039*	
C33	0.2330 (3)	0.2398 (3)	0.5059 (2)	0.0352 (7)	
H33A	0.2503	0.2138	0.5815	0.042*	
H33B	0.1951	0.3115	0.4826	0.042*	
C34	0.1442 (3)	0.1534 (2)	0.5075 (2)	0.0317 (7)	
H34	0.0653	0.1424	0.5605	0.038*	
C35	0.2028 (3)	0.0416 (2)	0.5449 (2)	0.0343 (7)	
H35A	0.2189	0.0135	0.6211	0.041*	
H35B	0.1453	−0.0145	0.5458	0.041*	
C36	0.3239 (3)	0.0588 (2)	0.4647 (2)	0.0298 (7)	
H36	0.3627	−0.0135	0.4893	0.036*	
C37	0.2961 (2)	0.0997 (2)	0.3474 (2)	0.0248 (6)	
H37A	0.3739	0.1095	0.2949	0.030*	
H37B	0.2392	0.0430	0.3486	0.030*	
C38	0.4127 (2)	0.1466 (2)	0.4617 (2)	0.0312 (7)	
H38A	0.4319	0.1205	0.5366	0.037*	
H38B	0.4910	0.1575	0.4095	0.037*	
C39	0.1164 (2)	0.1944 (2)	0.3902 (2)	0.0265 (6)	
H39A	0.0577	0.1388	0.3916	0.032*	
H39B	0.0776	0.2658	0.3659	0.032*	
C40	−0.2808 (2)	−0.3360 (2)	0.1403 (2)	0.0265 (6)	
H40A	−0.3270	−0.3987	0.1428	0.040*	
H40B	−0.3021	−0.2655	0.0819	0.040*	
H40C	−0.3023	−0.3377	0.2123	0.040*	

### Atomic displacement parameters (Å^2^)

**Table:**

	*U*^11^	*U*^22^	*U*^33^	*U*^12^	*U*^13^	*U*^23^
S1	0.0177 (4)	0.0212 (4)	0.0290 (4)	0.0032 (3)	−0.0085 (3)	−0.0108 (3)
O1	0.0208 (11)	0.0255 (11)	0.0340 (11)	0.0054 (8)	−0.0092 (8)	−0.0119 (9)
O2	0.0232 (11)	0.0238 (11)	0.0607 (14)	0.0043 (8)	−0.0137 (10)	−0.0241 (11)
N1	0.0162 (12)	0.0213 (12)	0.0255 (12)	0.0040 (9)	−0.0062 (9)	−0.0108 (10)
N2	0.0159 (12)	0.0215 (12)	0.0275 (13)	0.0032 (9)	−0.0069 (10)	−0.0123 (11)
C1	0.0142 (13)	0.0246 (15)	0.0218 (14)	0.0024 (11)	−0.0028 (10)	−0.0117 (12)
C2	0.0166 (14)	0.0218 (14)	0.0252 (15)	0.0002 (11)	0.0006 (11)	−0.0131 (12)
C3	0.0175 (14)	0.0235 (15)	0.0343 (16)	0.0065 (11)	−0.0065 (12)	−0.0128 (13)
C4	0.0239 (15)	0.0207 (15)	0.0415 (18)	0.0052 (12)	−0.0044 (13)	−0.0145 (14)
C5	0.0189 (15)	0.0258 (16)	0.0366 (17)	0.0008 (11)	−0.0022 (12)	−0.0202 (14)
C6	0.0187 (14)	0.0245 (15)	0.0287 (15)	0.0029 (11)	−0.0044 (11)	−0.0142 (13)
C7	0.0166 (14)	0.0222 (14)	0.0253 (15)	0.0035 (11)	−0.0016 (11)	−0.0123 (12)
C8	0.0205 (15)	0.0215 (14)	0.0231 (14)	0.0056 (11)	−0.0062 (11)	−0.0129 (12)
C9	0.0231 (15)	0.0223 (15)	0.0323 (16)	0.0058 (11)	−0.0095 (12)	−0.0177 (13)
C10	0.0185 (14)	0.0204 (14)	0.0288 (15)	0.0031 (11)	−0.0057 (11)	−0.0130 (12)
C11	0.0226 (15)	0.0213 (15)	0.0371 (17)	0.0031 (11)	−0.0122 (12)	−0.0152 (13)
C12	0.0190 (15)	0.0248 (16)	0.0458 (18)	−0.0010 (11)	−0.0070 (13)	−0.0158 (14)
C13	0.0255 (16)	0.0283 (17)	0.0342 (17)	0.0001 (12)	−0.0035 (13)	−0.0094 (14)
C14	0.0241 (16)	0.0334 (17)	0.0263 (16)	0.0020 (12)	−0.0088 (12)	−0.0094 (14)
C15	0.0351 (19)	0.053 (2)	0.0327 (17)	−0.0077 (15)	0.0019 (14)	−0.0264 (16)
C16	0.0288 (17)	0.0324 (17)	0.0457 (19)	0.0024 (13)	0.0027 (14)	−0.0241 (16)
C17	0.0210 (15)	0.0239 (15)	0.0426 (18)	0.0030 (12)	−0.0054 (12)	−0.0196 (14)
C18	0.0191 (16)	0.0361 (18)	0.0429 (19)	0.0003 (13)	0.0001 (13)	−0.0163 (15)
C19	0.0207 (15)	0.0254 (15)	0.0297 (16)	0.0035 (11)	−0.0104 (12)	−0.0132 (13)
C20	0.0215 (16)	0.0326 (17)	0.0452 (18)	0.0015 (12)	−0.0085 (13)	−0.0244 (15)
S2	0.0164 (4)	0.0229 (4)	0.0257 (4)	0.0029 (3)	−0.0058 (3)	−0.0146 (3)
O3	0.0173 (10)	0.0285 (11)	0.0343 (11)	0.0055 (8)	−0.0073 (8)	−0.0196 (9)
O4	0.0214 (11)	0.0266 (11)	0.0414 (12)	−0.0012 (8)	−0.0064 (8)	−0.0224 (10)
N3	0.0173 (12)	0.0215 (12)	0.0200 (12)	0.0045 (9)	−0.0068 (9)	−0.0105 (10)
N4	0.0137 (11)	0.0224 (12)	0.0279 (13)	0.0015 (10)	−0.0055 (10)	−0.0138 (11)
C21	0.0190 (14)	0.0183 (14)	0.0178 (13)	0.0048 (10)	−0.0089 (10)	−0.0095 (11)
C22	0.0196 (14)	0.0189 (14)	0.0184 (13)	0.0017 (11)	−0.0077 (11)	−0.0073 (11)
C23	0.0174 (14)	0.0311 (16)	0.0245 (15)	0.0078 (11)	−0.0066 (11)	−0.0158 (13)
C24	0.0254 (15)	0.0228 (14)	0.0268 (15)	0.0046 (11)	−0.0065 (11)	−0.0174 (13)
C25	0.0221 (15)	0.0226 (15)	0.0245 (15)	−0.0008 (11)	−0.0067 (11)	−0.0100 (12)
C26	0.0143 (14)	0.0234 (15)	0.0231 (14)	0.0033 (11)	−0.0031 (11)	−0.0097 (12)
C27	0.0210 (14)	0.0172 (13)	0.0206 (14)	0.0032 (10)	−0.0074 (11)	−0.0103 (11)
C28	0.0203 (15)	0.0190 (14)	0.0217 (14)	0.0057 (11)	−0.0055 (11)	−0.0094 (12)
C29	0.0215 (15)	0.0184 (14)	0.0286 (15)	0.0025 (11)	−0.0070 (11)	−0.0114 (12)
C30	0.0156 (14)	0.0206 (14)	0.0306 (15)	0.0036 (10)	−0.0078 (11)	−0.0133 (12)
C31	0.0230 (15)	0.0275 (16)	0.0367 (17)	0.0024 (12)	−0.0107 (12)	−0.0192 (14)
C32	0.0266 (17)	0.0415 (18)	0.0403 (18)	0.0022 (13)	−0.0118 (13)	−0.0276 (16)
C33	0.0354 (18)	0.0459 (19)	0.0395 (18)	0.0121 (14)	−0.0155 (14)	−0.0300 (16)
C34	0.0227 (16)	0.0449 (19)	0.0338 (17)	0.0048 (13)	−0.0020 (12)	−0.0250 (15)
C35	0.0372 (19)	0.0398 (19)	0.0252 (16)	−0.0032 (14)	−0.0090 (13)	−0.0142 (14)
C36	0.0305 (17)	0.0292 (16)	0.0323 (16)	0.0104 (13)	−0.0127 (13)	−0.0149 (14)
C37	0.0223 (15)	0.0266 (15)	0.0301 (16)	0.0054 (12)	−0.0066 (12)	−0.0167 (13)
C38	0.0213 (16)	0.0486 (19)	0.0311 (16)	0.0079 (13)	−0.0122 (12)	−0.0227 (15)
C39	0.0197 (15)	0.0333 (16)	0.0334 (16)	0.0050 (12)	−0.0070 (12)	−0.0211 (14)
C40	0.0205 (15)	0.0250 (15)	0.0344 (16)	−0.0008 (11)	−0.0077 (12)	−0.0139 (13)

### Geometric parameters (Å, º)

**Table:**

S1—C7	1.739 (3)	S2—C21	1.742 (2)
S1—C1	1.756 (2)	S2—C27	1.743 (3)
O1—C8	1.225 (3)	O3—C28	1.223 (3)
O2—C5	1.368 (3)	O4—C25	1.372 (3)
O2—C20	1.436 (3)	O4—C40	1.429 (3)
N1—C1	1.299 (3)	N3—C21	1.300 (3)
N1—C2	1.401 (3)	N3—C22	1.409 (3)
N2—C8	1.374 (3)	N4—C28	1.376 (3)
N2—C1	1.385 (3)	N4—C21	1.391 (3)
N2—H2N	0.87 (3)	N4—H4N	0.78 (3)
C2—C7	1.397 (3)	C22—C23	1.396 (3)
C2—C3	1.399 (3)	C22—C27	1.403 (3)
C3—C4	1.381 (4)	C23—C24	1.372 (3)
C3—H3	0.9500	C23—H23	0.9500
C4—C5	1.404 (4)	C24—C25	1.401 (3)
C4—H4	0.9500	C24—H24	0.9500
C5—C6	1.389 (4)	C25—C26	1.389 (4)
C6—C7	1.398 (3)	C26—C27	1.397 (3)
C6—H6	0.9500	C26—H26	0.9500
C8—C9	1.506 (3)	C28—C29	1.502 (3)
C9—C10	1.542 (4)	C29—C30	1.556 (3)
C9—H9A	0.9900	C29—H29A	0.9900
C9—H9B	0.9900	C29—H29B	0.9900
C10—C11	1.537 (3)	C30—C39	1.538 (3)
C10—C17	1.539 (3)	C30—C37	1.539 (3)
C10—C19	1.543 (3)	C30—C31	1.540 (3)
C11—C12	1.530 (4)	C31—C32	1.539 (4)
C11—H11A	0.9900	C31—H31A	0.9900
C11—H11B	0.9900	C31—H31B	0.9900
C12—C13	1.532 (4)	C32—C38	1.523 (4)
C12—C18	1.534 (4)	C32—C33	1.533 (4)
C12—H12	1.0000	C32—H32	1.0000
C13—C14	1.534 (4)	C33—C34	1.526 (4)
C13—H13A	0.9900	C33—H33A	0.9900
C13—H13B	0.9900	C33—H33B	0.9900
C14—C15	1.528 (4)	C34—C35	1.540 (4)
C14—C19	1.528 (4)	C34—C39	1.540 (4)
C14—H14	1.0000	C34—H34	1.0000
C15—C16	1.529 (4)	C35—C36	1.526 (4)
C15—H15A	0.9900	C35—H35A	0.9900
C15—H15B	0.9900	C35—H35B	0.9900
C16—C18	1.534 (4)	C36—C38	1.532 (4)
C16—C17	1.538 (4)	C36—C37	1.540 (4)
C16—H16	1.0000	C36—H36	1.0000
C17—H17A	0.9900	C37—H37A	0.9900
C17—H17B	0.9900	C37—H37B	0.9900
C18—H18A	0.9900	C38—H38A	0.9900
C18—H18B	0.9900	C38—H38B	0.9900
C19—H19A	0.9900	C39—H39A	0.9900
C19—H19B	0.9900	C39—H39B	0.9900
C20—H20A	0.9800	C40—H40A	0.9800
C20—H20B	0.9800	C40—H40B	0.9800
C20—H20C	0.9800	C40—H40C	0.9800
			
C7—S1—C1	87.98 (12)	C21—S2—C27	88.08 (12)
C5—O2—C20	117.2 (2)	C25—O4—C40	117.6 (2)
C1—N1—C2	109.1 (2)	C21—N3—C22	108.6 (2)
C8—N2—C1	123.1 (2)	C28—N4—C21	124.1 (2)
C8—N2—H2N	116.6 (17)	C28—N4—H4N	117 (2)
C1—N2—H2N	119.9 (17)	C21—N4—H4N	119 (2)
N1—C1—N2	122.0 (2)	N3—C21—N4	120.3 (2)
N1—C1—S1	117.39 (19)	N3—C21—S2	118.23 (19)
N2—C1—S1	120.53 (19)	N4—C21—S2	121.46 (19)
C7—C2—C3	118.8 (2)	C23—C22—C27	118.9 (2)
C7—C2—N1	115.4 (2)	C23—C22—N3	125.9 (2)
C3—C2—N1	125.7 (2)	C27—C22—N3	115.3 (2)
C4—C3—C2	119.2 (2)	C24—C23—C22	119.1 (2)
C4—C3—H3	120.4	C24—C23—H23	120.4
C2—C3—H3	120.4	C22—C23—H23	120.4
C3—C4—C5	121.2 (3)	C23—C24—C25	121.6 (2)
C3—C4—H4	119.4	C23—C24—H24	119.2
C5—C4—H4	119.4	C25—C24—H24	119.2
O2—C5—C6	124.1 (2)	O4—C25—C26	124.9 (2)
O2—C5—C4	115.1 (2)	O4—C25—C24	114.3 (2)
C6—C5—C4	120.8 (2)	C26—C25—C24	120.8 (2)
C5—C6—C7	117.1 (2)	C25—C26—C27	117.0 (2)
C5—C6—H6	121.4	C25—C26—H26	121.5
C7—C6—H6	121.4	C27—C26—H26	121.5
C2—C7—C6	122.9 (2)	C26—C27—C22	122.7 (2)
C2—C7—S1	110.02 (19)	C26—C27—S2	127.5 (2)
C6—C7—S1	126.9 (2)	C22—C27—S2	109.81 (18)
O1—C8—N2	121.3 (2)	O3—C28—N4	120.8 (2)
O1—C8—C9	123.1 (2)	O3—C28—C29	123.6 (2)
N2—C8—C9	115.5 (2)	N4—C28—C29	115.6 (2)
C8—C9—C10	112.4 (2)	C28—C29—C30	113.0 (2)
C8—C9—H9A	109.1	C28—C29—H29A	109.0
C10—C9—H9A	109.1	C30—C29—H29A	109.0
C8—C9—H9B	109.1	C28—C29—H29B	109.0
C10—C9—H9B	109.1	C30—C29—H29B	109.0
H9A—C9—H9B	107.9	H29A—C29—H29B	107.8
C11—C10—C17	108.7 (2)	C39—C30—C37	108.9 (2)
C11—C10—C9	108.1 (2)	C39—C30—C31	108.6 (2)
C17—C10—C9	111.7 (2)	C37—C30—C31	108.8 (2)
C11—C10—C19	108.2 (2)	C39—C30—C29	111.3 (2)
C17—C10—C19	108.9 (2)	C37—C30—C29	111.3 (2)
C9—C10—C19	111.2 (2)	C31—C30—C29	107.9 (2)
C12—C11—C10	110.6 (2)	C32—C31—C30	110.2 (2)
C12—C11—H11A	109.5	C32—C31—H31A	109.6
C10—C11—H11A	109.5	C30—C31—H31A	109.6
C12—C11—H11B	109.5	C32—C31—H31B	109.6
C10—C11—H11B	109.5	C30—C31—H31B	109.6
H11A—C11—H11B	108.1	H31A—C31—H31B	108.1
C11—C12—C13	110.0 (2)	C38—C32—C33	109.5 (2)
C11—C12—C18	109.1 (2)	C38—C32—C31	109.3 (2)
C13—C12—C18	109.1 (2)	C33—C32—C31	109.4 (2)
C11—C12—H12	109.6	C38—C32—H32	109.5
C13—C12—H12	109.6	C33—C32—H32	109.5
C18—C12—H12	109.6	C31—C32—H32	109.5
C12—C13—C14	109.3 (2)	C34—C33—C32	109.4 (2)
C12—C13—H13A	109.8	C34—C33—H33A	109.8
C14—C13—H13A	109.8	C32—C33—H33A	109.8
C12—C13—H13B	109.8	C34—C33—H33B	109.8
C14—C13—H13B	109.8	C32—C33—H33B	109.8
H13A—C13—H13B	108.3	H33A—C33—H33B	108.2
C15—C14—C19	109.4 (2)	C33—C34—C35	109.7 (2)
C15—C14—C13	109.2 (2)	C33—C34—C39	109.8 (2)
C19—C14—C13	109.8 (2)	C35—C34—C39	109.2 (2)
C15—C14—H14	109.5	C33—C34—H34	109.4
C19—C14—H14	109.5	C35—C34—H34	109.4
C13—C14—H14	109.5	C39—C34—H34	109.4
C14—C15—C16	109.9 (2)	C36—C35—C34	109.4 (2)
C14—C15—H15A	109.7	C36—C35—H35A	109.8
C16—C15—H15A	109.7	C34—C35—H35A	109.8
C14—C15—H15B	109.7	C36—C35—H35B	109.8
C16—C15—H15B	109.7	C34—C35—H35B	109.8
H15A—C15—H15B	108.2	H35A—C35—H35B	108.3
C15—C16—C18	109.5 (2)	C35—C36—C38	110.1 (2)
C15—C16—C17	109.5 (2)	C35—C36—C37	108.9 (2)
C18—C16—C17	108.8 (2)	C38—C36—C37	109.1 (2)
C15—C16—H16	109.7	C35—C36—H36	109.6
C18—C16—H16	109.7	C38—C36—H36	109.6
C17—C16—H16	109.7	C37—C36—H36	109.6
C16—C17—C10	110.2 (2)	C30—C37—C36	110.3 (2)
C16—C17—H17A	109.6	C30—C37—H37A	109.6
C10—C17—H17A	109.6	C36—C37—H37A	109.6
C16—C17—H17B	109.6	C30—C37—H37B	109.6
C10—C17—H17B	109.6	C36—C37—H37B	109.6
H17A—C17—H17B	108.1	H37A—C37—H37B	108.1
C16—C18—C12	109.8 (2)	C32—C38—C36	109.9 (2)
C16—C18—H18A	109.7	C32—C38—H38A	109.7
C12—C18—H18A	109.7	C36—C38—H38A	109.7
C16—C18—H18B	109.7	C32—C38—H38B	109.7
C12—C18—H18B	109.7	C36—C38—H38B	109.7
H18A—C18—H18B	108.2	H38A—C38—H38B	108.2
C14—C19—C10	110.4 (2)	C30—C39—C34	109.9 (2)
C14—C19—H19A	109.6	C30—C39—H39A	109.7
C10—C19—H19A	109.6	C34—C39—H39A	109.7
C14—C19—H19B	109.6	C30—C39—H39B	109.7
C10—C19—H19B	109.6	C34—C39—H39B	109.7
H19A—C19—H19B	108.1	H39A—C39—H39B	108.2
O2—C20—H20A	109.5	O4—C40—H40A	109.5
O2—C20—H20B	109.5	O4—C40—H40B	109.5
H20A—C20—H20B	109.5	H40A—C40—H40B	109.5
O2—C20—H20C	109.5	O4—C40—H40C	109.5
H20A—C20—H20C	109.5	H40A—C40—H40C	109.5
H20B—C20—H20C	109.5	H40B—C40—H40C	109.5
			
C2—N1—C1—N2	178.0 (2)	C22—N3—C21—N4	179.9 (2)
C2—N1—C1—S1	0.4 (3)	C22—N3—C21—S2	0.2 (3)
C8—N2—C1—N1	179.2 (2)	C28—N4—C21—N3	−174.2 (2)
C8—N2—C1—S1	−3.2 (3)	C28—N4—C21—S2	5.6 (3)
C7—S1—C1—N1	0.8 (2)	C27—S2—C21—N3	−0.4 (2)
C7—S1—C1—N2	−176.8 (2)	C27—S2—C21—N4	179.9 (2)
C1—N1—C2—C7	−1.9 (3)	C21—N3—C22—C23	−179.6 (2)
C1—N1—C2—C3	175.1 (2)	C21—N3—C22—C27	0.2 (3)
C7—C2—C3—C4	−0.7 (4)	C27—C22—C23—C24	0.4 (4)
N1—C2—C3—C4	−177.7 (2)	N3—C22—C23—C24	−179.9 (2)
C2—C3—C4—C5	0.5 (4)	C22—C23—C24—C25	1.1 (4)
C20—O2—C5—C6	7.2 (4)	C40—O4—C25—C26	2.8 (4)
C20—O2—C5—C4	−171.8 (2)	C40—O4—C25—C24	−177.7 (2)
C3—C4—C5—O2	179.1 (2)	C23—C24—C25—O4	178.5 (2)
C3—C4—C5—C6	0.2 (4)	C23—C24—C25—C26	−2.0 (4)
O2—C5—C6—C7	−179.4 (2)	O4—C25—C26—C27	−179.2 (2)
C4—C5—C6—C7	−0.6 (4)	C24—C25—C26—C27	1.3 (4)
C3—C2—C7—C6	0.3 (4)	C25—C26—C27—C22	0.1 (4)
N1—C2—C7—C6	177.6 (2)	C25—C26—C27—S2	179.76 (19)
C3—C2—C7—S1	−174.76 (19)	C23—C22—C27—C26	−1.0 (4)
N1—C2—C7—S1	2.5 (3)	N3—C22—C27—C26	179.3 (2)
C5—C6—C7—C2	0.4 (4)	C23—C22—C27—S2	179.33 (19)
C5—C6—C7—S1	174.5 (2)	N3—C22—C27—S2	−0.4 (3)
C1—S1—C7—C2	−1.78 (19)	C21—S2—C27—C26	−179.3 (2)
C1—S1—C7—C6	−176.6 (2)	C21—S2—C27—C22	0.41 (19)
C1—N2—C8—O1	−7.3 (4)	C21—N4—C28—O3	−4.5 (4)
C1—N2—C8—C9	168.9 (2)	C21—N4—C28—C29	172.7 (2)
O1—C8—C9—C10	75.8 (3)	O3—C28—C29—C30	80.6 (3)
N2—C8—C9—C10	−100.3 (3)	N4—C28—C29—C30	−96.5 (3)
C8—C9—C10—C11	174.6 (2)	C28—C29—C30—C39	−63.1 (3)
C8—C9—C10—C17	55.1 (3)	C28—C29—C30—C37	58.5 (3)
C8—C9—C10—C19	−66.8 (3)	C28—C29—C30—C31	177.8 (2)
C17—C10—C11—C12	−59.1 (3)	C39—C30—C31—C32	59.5 (3)
C9—C10—C11—C12	179.5 (2)	C37—C30—C31—C32	−58.9 (3)
C19—C10—C11—C12	59.0 (3)	C29—C30—C31—C32	−179.7 (2)
C10—C11—C12—C13	−59.8 (3)	C30—C31—C32—C38	60.0 (3)
C10—C11—C12—C18	59.7 (3)	C30—C31—C32—C33	−60.0 (3)
C11—C12—C13—C14	59.0 (3)	C38—C32—C33—C34	−60.1 (3)
C18—C12—C13—C14	−60.6 (3)	C31—C32—C33—C34	59.7 (3)
C12—C13—C14—C15	60.8 (3)	C32—C33—C34—C35	60.0 (3)
C12—C13—C14—C19	−59.2 (3)	C32—C33—C34—C39	−60.0 (3)
C19—C14—C15—C16	60.0 (3)	C33—C34—C35—C36	−59.4 (3)
C13—C14—C15—C16	−60.2 (3)	C39—C34—C35—C36	61.0 (3)
C14—C15—C16—C18	59.4 (3)	C34—C35—C36—C38	58.7 (3)
C14—C15—C16—C17	−59.8 (3)	C34—C35—C36—C37	−60.9 (3)
C15—C16—C17—C10	59.4 (3)	C39—C30—C37—C36	−59.3 (3)
C18—C16—C17—C10	−60.2 (3)	C31—C30—C37—C36	58.9 (3)
C11—C10—C17—C16	59.2 (3)	C29—C30—C37—C36	177.7 (2)
C9—C10—C17—C16	178.4 (2)	C35—C36—C37—C30	60.5 (3)
C19—C10—C17—C16	−58.4 (3)	C38—C36—C37—C30	−59.7 (3)
C15—C16—C18—C12	−59.2 (3)	C33—C32—C38—C36	59.6 (3)
C17—C16—C18—C12	60.4 (3)	C31—C32—C38—C36	−60.3 (3)
C11—C12—C18—C16	−60.2 (3)	C35—C36—C38—C32	−59.3 (3)
C13—C12—C18—C16	59.9 (3)	C37—C36—C38—C32	60.1 (3)
C15—C14—C19—C10	−59.7 (3)	C37—C30—C39—C34	59.0 (3)
C13—C14—C19—C10	60.1 (3)	C31—C30—C39—C34	−59.3 (3)
C11—C10—C19—C14	−59.2 (3)	C29—C30—C39—C34	−178.0 (2)
C17—C10—C19—C14	58.8 (3)	C33—C34—C39—C30	60.2 (3)
C9—C10—C19—C14	−177.7 (2)	C35—C34—C39—C30	−60.1 (3)

### Hydrogen-bond geometry (Å, º)

**Table:**

*D*—H···*A*	*D*—H	H···*A*	*D*···*A*	*D*—H···*A*
N2—H2*N*···N3	0.87 (3)	2.13 (3)	2.995 (3)	169 (2)
N4—H4*N*···N1	0.78 (3)	2.30 (3)	3.077 (3)	174 (2)
C6—H6···O3^i^	0.95	2.58	3.452 (3)	153
C26—H26···O1^ii^	0.95	2.45	3.392 (3)	174

Symmetry codes: (i) *x*+1, *y*, *z*; (ii) *x*−1, *y*, *z*.
